# Fly Stampede 2.0: A Next Generation Optomotor Assay for Walking Behavior in *Drosophila Melanogaster*

**DOI:** 10.3389/fnmol.2016.00148

**Published:** 2016-12-27

**Authors:** Soomin Kim, Kelly Tellez, Graham Buchan, Tim Lebestky

**Affiliations:** Department of Biology, Williams CollegeWilliamstown, MA, USA

**Keywords:** dopamine, dopamine receptor, arousal, attention, *Drosophila*, behavior, optomotor, visual perception

## Abstract

Optomotor behavior represents a stereotyped locomotor response to visual motion that is found in both vertebrate and invertebrate models. The Fly Stampede assay was developed to study an optomotor response in freely walking populations of *Drosophila*. Here we share optimized assay designs and software for production of a modified stampede assay that can be used for genetic screens, and improved tracking outputs for understanding behavioral parameters of visual-motion responses and arousal state of individual animals. Arousal state influences behavioral performance in the stampede assay. As proof of principle experiments we show parametric modulation of visual stimuli and startle stimuli in both wildtype and mutant flies for the type I family dopamine receptor Dop1R1 (DopR). *DopR* mutants are hyperactive and perform poorly in the stampede assay, suggesting a potential role in visual perception and/or arousal. The stampede assay creates an efficient platform for rapid screening of mutant animals or circuit manipulations for investigating attentional processes in *Drosophila*.

## Introduction

Locomotor responses to moving visual stimuli in conscious animals have been studied extensively in vertebrate and invertebrate model systems (reviewed in Portugues and Engert, 2009; Borst et al., 2010; Gohl et al., 2012; Kretschmer et al., 2015). The perception of visual information in driving the trajectory and speed of a behavioral response has been a rich vein of research for understanding information processing in the brain and also the relationship between internal and external modulators of behavioral responses. In *Drosophila melanogaster*, the optomotor response has been utilized in both terrestrial walking assays as well as navigation in tethered and free flight (David, 1979; Strauss et al., 1997; Wolf and Heisenberg, 1990). While traditional assays focused on the study of individual behaviors, optomotor responses can also be studied in populations of freely moving animals (Tammero et al., 2004). The Frye lab at UCLA engineered a straightforward, elegant optomotor chamber to study the optomotor response of fly populations, dubbed the “Fly Stampede” that was used to characterize brain circuitry that separates visual motion cues from phototaxis (Zhu et al., 2009). The assay was used to follow the spatial distribution of 100 moving flies in a long square tube, or arena, as they respond to either visual motion as represented by LED panels on a three-sided visual “hallway” that toggles a “centering” motion stimulus that drives the animals to the middle of the arena, or an “ends” motion stimulus that drives the animals to both termini of the arena. The strength of the behavioral response observed was dependent on the number of flies included in an assay, and higher fidelity of behavioral responses to the visual motion were recorded for larger numbers of animals used (personal communication). This observation may be partially explained by recent evidence highlighting the relationship between social mechanosensory contact and herd-like aversive walking behavior (Suh et al., 2004; Ramdya et al., 2015). This may suggest that optomotor behavior in the stampede assay can be reinforced or modulated by group behavior, making it essential to be aware of the underlying sensory mechanisms at play and the necessity of parallel experiments for individual (single-fly) behavioral assays.

A separate parameter of the stampede assay that significantly influences behavioral performance is the underlying arousal state of the animals when presenting the visual motion stimulus. The stampede chamber utilizes small vibrating motors mounted near each end of the walking arena to generate a mechanical startle event prior to presentation of visual motion stimulus. In the absence of startle, the animals perform worse in the optomotor response (personal communication, Frye and Zhu). The dopamine receptor Dop1R1 (DopR) has been shown to be involved in negative regulation or dampening responses for arousal states generated by air puff startle (Lebestky et al., 2009). Thus, we also wanted to assess whether the stampede assay would show optomotor differences when modulating mechanosensory startle in the stampede assay.

While the original fly stampede assay was informative for preliminary investigation of the relationship between arousal and optomotor response, it also presented some experimental limitations. The visualization of the flies was restricted by infrared side-lighting at the termini that creates “hot spots” of high illumination at the distal ends of the arena that partially obscure fly behavior at the termini, making it difficult to accurately track individual animals discretely for generation of speed or trajectory data. This also limited the types of visual motion patterns that could be utilized for testing behavior, as a centering stimulus that brought animals to the middle of the arena was optimal for capturing the majority of flies instead of patterns to drive animals from one side of the arena to the other. The original design also placed startle motors in close contact to the LED panels used for generating visual patterns. After many repeated trials, the shaking stimulus may loosen or break circuit linkages for the LEDs and occasionally led to unpredictable failures in the visual stimulus during experimental trials. Additionally, the original assay uses a singular arena, making it harder to execute intermediate scale genetic or circuit activation screens that may require higher behavioral trial throughput.

In the following sections, we will describe our changes to the original assay and share all design blueprints and software for production of the new stampede arena (supplementary data). Any lab with access to a laser cutter can easily duplicate or modify our design plans for the arena platform, and circuit board designs for mounting LED panels and the Infrared lighting source for visualizing *Drosophila* behavior are also included. We include the python BIAS software program that coordinates visual motion stimuli patterns, the mechanical startle stimuli, and camera recording. BIAS can run on Linux or Windows operating system platforms. We highlight the parametric characterization of the visual stimuli as well as the startle stimuli for individual (single-fly) experiments using wildtype *Drosophila* in our arena. As proof of principle, we present a genotypic comparison between wildtype animals and a strong hypomorphic allele in the Type I dopamine receptor (*DopR^f02676^*). While our stampede assay can be used in the same way as the original assay to monitor population behavior, all phenotypic results presented here are based on comparing behavioral trials of individual flies to isolate the relationship between arousal and optomotor behavior. This removes confounding variables associated with social behaviors within population-based experiments. Our data suggests a role for dopamine in the stampede behavior, and highlights the potential use of our arena design for genetic or circuit manipulation screens for identifying substrates and molecules involved in arousal and attentional processes.

## Materials and Methods

### Stampede Design and Software Use

All design files and software are available in the supplementary materials. These designs were commissioned using ioRodeo, an independent engineering firm specializing in hardware/software interface. The BIAS software program coordinates the linkage between the LED Panel Controller, Motor Control for Startle Stimulus, and the camera (Point Gray) to record behavior. The program records a digital movie file for post-processing using the experimenter’s preferred tracking program. Our data is recorded as .avi files and analyzed by Ethovision XT software. Although not utilized for this article, we also include our tracking software for median population trajectory in supplementary materials.

### *Drosophila* Stocks

The wildtype stock used for all experiments is *Canton-S* *(CS)*. The dominant hypomorphic mutant allele *DopR^f02676^* contains a *piggy-Bac* element insertion with a UAS sequence in the first intron (Exelixis Collection at Harvard medical School). The *DopR^f02676^* allele was backcrossed into the *CS* wild-type background for six generations (Pitmon et al., 2016). Fly stocks were maintained at 18°C, and crosses were grown at 25°C. All flies were kept in 12:12 light:dark cycle conditions. Flies were reared on Bloomington recipe fly food.

### Stampede Assays

Three to five day old males were collected in batches of 10 animals and stored overnight in vials at 25°C and maintained on the same 12:12 Light/Dark cycle (8 AM–8 PM). The temperature in the behavioral room ranged from 22°C to 24°C with 40%–60% humidity. All experiments were performed between 12:00 PM and 5 PM. Individual males are aspirated into small 5 mm diameter tubes^1^ mounted on the arena platform in contact with the startle motors. Flies acclimate to tubes for 5 min prior to performing the assay. It is essential that the assays are performed in a dark room with no overhead lighting or ambient light from computer monitors or other equipment that will affect the brightness/contrast of the presented visual motion stimulus. Black Hardboard (Thor labs) was mounted around the behavioral chamber to limit ambient light exposure from the nearby computer screen.

The standard assay conditions measure flies’ responses to the following ordered events: mechanical startle (3 s) > visual motion left (1 min) > mechanical startle (3 s) > visual motion right (1 min) > mechanical startle (3 s) > no visual motion (1 min). The startle stimulus is produced by activating the vibrating motors, mounted at each end of the tube (2 discrete events duration 1 s each with a 1 s interval). This is followed by 1 min of visual motion (25 Hz rate) in which two columns of LED bulbs within an individual panel (green vertical stripe) sweep across the visual field in one direction (left). This is followed by an identical startle stimulus (as described above), followed by 1 min of visual motion in the opposite direction at the same rate (right). A final startle stimulus is triggered and 1 min of locomotor behavior is recorded in the absence of visual stimuli.

All parameters of the timing and activation of individual startle events, the firing rate, pattern and duration of visual motion stimuli through the LED panel hallway, and the triggering and acquisition of camera footage are controlled by the Graphic User Interface within the BIAS program (supplementary data). This integration of all stimuli parameters and their recording by the camera is controlled in part by configuration files that define the ordering of events and the pattern and speed (Example included for 25 Hz LED motion stimulus with both the buzz and no buzz configurations used as the standard assay found in supplementary materials). Timing of the triggering and duration of filming is controlled within the GUI, and the live feed of the camera is present for monitoring within the GUI window during the performance of the assay. We have included a basic user protocol as well as a generic configuration file that can be easily modified to suit alternate experimental needs within the supplementary materials.

## Results

**Figure 1:**
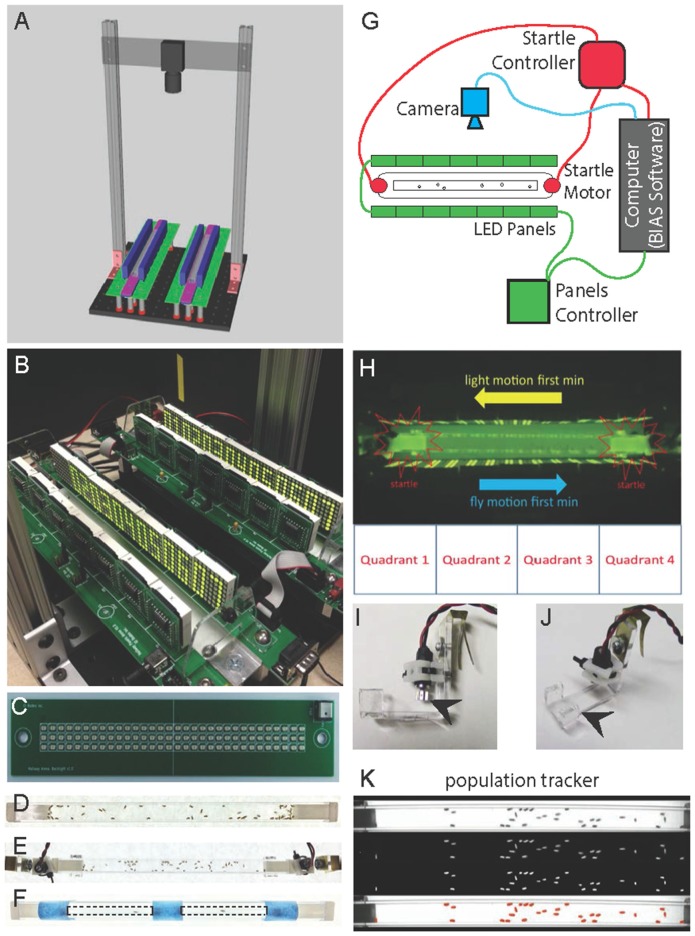
**Fly Stampede 2.0. (A)** Schematic drawing of the Fly Stampede. All individual components are available as pyCAD files for production (Supplementary materials). **(B)** Close view of completed Stampede with LED Panels and IR Backlight/Diffusion Plate. **(C)** Close view of infrared Backlight LED Array for visualizing flies. The plate is mounted under a diffuser plate and points upwards to illuminate the entire region and discriminate fly locomotion without utilizing natural light. **(D)** Top-down view of 50 flies in population tube. **(E)** Top-down view of 50 flies in population tube/arena in clamps with associated motors. **(F)** Top-down view of individual fly arenas (2 mm × 5 mm diameter) for stampede assay. Dashed lines indicate boundaries of individual fly tubes mounted on top of the standard population tubes (blue tape). **(G)** BIAS software coordinates the triggering, order and duration of LED panels (green), the startle motors (red) and the camera (blue). **(H)** Flies move in the opposite direction of the visual motion. The visual motion runs in one direction for 1 min and then reverses in the opposite direction for the second minute. Startle motors are mounted at the distal ends of the tube holding the flies. The tracking software can separate the tube into four equal quadrants to determine % time occupancy reflecting appropriate optomotor responses to visual motion. **(I)** Side view of Startle Motors and the Mounting Clips to hold fly tubes in between the LED Panels (arrowhead marks oscillating motor). **(J)** Diagonal view of Startle Motors and the Mounting Clips to hold fly tubes in between the LED Panels (arrowhead marks acrylic clamp). **(K)** Median Centroids of the population of flies can be measured for populations, or discrete tracking of position, speed and trajectory for individual flies. Top panel (1) represents unfiltered image, next panel down (2) shows image after background subtraction, next panel (3) after assignment of centroids to flies as object, bottom panel (4) represents fidelity of tracked objects/flies.

**Figure 2:**
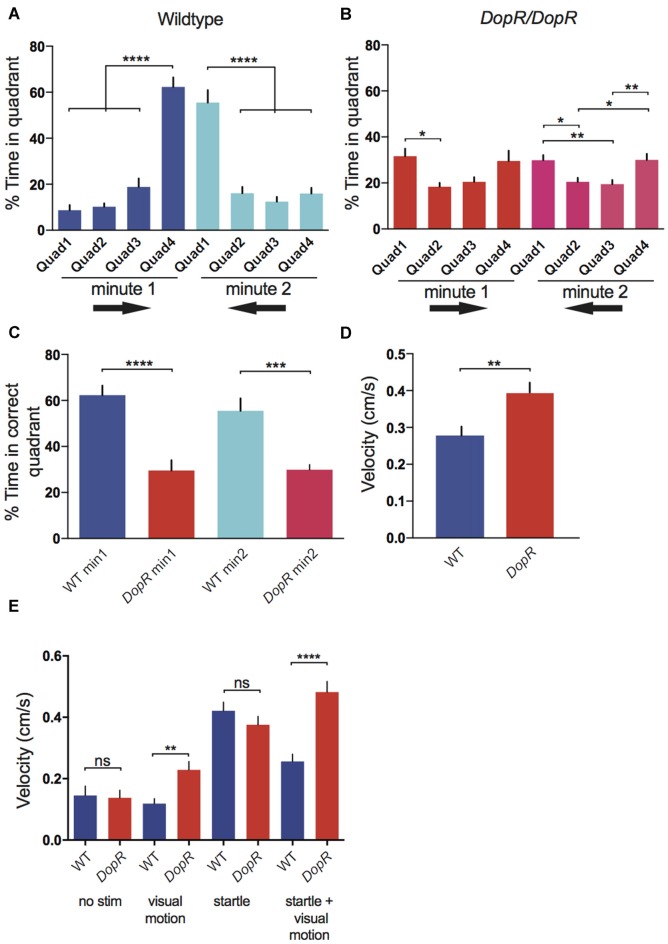
**Optomotor Response in wildtype and dopamine receptor Dop1R1 *(DopR)* mutant flies. (A)** Optomotor response as defined by % time spent in the correct quadrant. In the first minute, Quadrant 4 is the terminal optomotor target for flies, in the second minute Quadrant 1 is the target (optomotor response direction symbolized by black arrows). **(B)** Optomotor response in *DopR^f02676^/DopR^f02676^* homozygous mutant flies. **(C)** Comparison of optomotor response in wildtype and *DopR^f02676^/DopR^f02676^* homozygous mutant flies. **(D)** Velocity of wildtype and *DopR^f02676^/DopR^f02676^* mutant flies during the standard stampede assay condition. **(E)** Comparison of velocity for wildtype and DopR mutant flies in response to different assay conditions (no stimuli visual motion stimuli alone startle alone startle + visual motion). SEM represented for all conditions **(A–C)** *n* = 12 for all conditions. One-Way analysis of variance (ANOVA) with Bonferroni Correction. **(D,E)** *n* = 20 for all conditions. Paired *T*-tests **(A–E)** ****p < 0.0001 ***p < 0.001, **p < 0.01, *p < 0.05.

**Figure 3:**
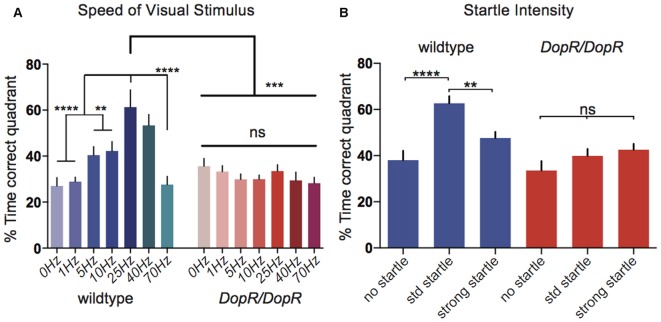
**Optomotor responses for modulated parameters of visual motion or startle stimuli. (A)** Comparison of optomotor response in wildtype and *DopR^f02676^/DopR^f02676^* homozygous mutant flies upon modulation of visual motion stimulus speed. *n* = 10 for all conditions. **(B)** Optomotor responses for wildtype flies upon modulation of startle parameters. *n* = 20 for all conditions. **(A,B)** One-Way ANOVA with Bonferroni Correction. ****p < 0.0001 ***p < 0.001, **p < 0.01.

The new fly stampede design allows for multiple improvements while capitalizing on the essential features of the original assay (Zhu et al., 2009). The new stampede allows simultaneous visualization of two separate arenas (Figures 1A,B). Mounting a single camera above the arenas can create independent tracking regions surrounding each individual arena, allowing for either two population trials or four individual fly trials (using 2 mm × 5 mm tubes per arena) per trial event. The infrared LED backlight array is mounted below the arenas, illuminating upwards through a white opaque diffusion plate that creates a uniform light source for tracking the entire arena region (Figure 1C). LED panels are mounted on individual circuit boards (Figure 1C) that are independent of the shaking motors mounted centrally on columns supporting the IR Backlights. Physically separating the LED panels from the shaking motors allows for more stable connectivity when generating the visual motion stimulus. It should be noted that the original assay does have an additional axis of LED panels that runs along the “floor” of the arena, meaning that the visual motion stimuli are presented to the animals from three sides, vs. two sides of presented motion in our assay. The assay can either be used for populations of animals (Figures 1D,E) or isolated individuals (Figure 1F). All data in Figures 2, 3 are based on single-fly experiments. The elicited behavior from responding to two sides of LED panels is robust and the advantages of visualizing the entire arena justify the loss of an axis of visual motion. We briefly tested a mirrored glass surface, mounted just above the IR diffusion plate, to more similarly recreate the 3-axis LED (side-side-underneath) that was utilized for the original assay (Zhu et al., 2009). The mirror allowed IR to pass and reflect the visual motion from LEDs. But, we did not see a robust difference in behavior relative to side-side stimuli patterns (data not shown).

The original software for triggering and recording the behavior was generated using the Matlab software suite (Mathworks) as the interface between the hardware, imaging, and data output for tracking the median population behavior. This programming platform eventually led to difficulties in running the trigger program due to incompatible upgrades and changes in the command language of Matlab. We have created a new integration program using python (BIAS program in supplementary materials) that controls the linkage between the LED panels (Figure 1G, green), mechanical startle (Figure 1G, red), and camera recording parameters (Figure 1G, blue). Through this program, experimenters can control the parameters of stimulus presentation (visual motion and mechanical startle) as well as the timing of video recording.

The standard assay used for our experiments uses visual motion to drive the optomotor response of animals to one side of the arena, before flipping the polarity of the response to the opposite side of the arena. A mechanical startle precedes the presentation of visual motion (Figure 1H). The fidelity of the animals’ optomotor performance is primarily measured by time of occupancy in the targeted region. For the standard assay, animals move towards or into quadrant 4 within the first minute, and move towards quadrant 1 in the second minute (Figure 1H). Mechanical startle is produced by symmetric vibrating motors (Figure 1I) that are mounted to each of the two plastic clamps that hold each end of the tube (Figure 1J). The tube is then held between the LED panels (Figure 1J). Movies are processed by either Ethovision XT for individual animals or by our median centroid population tracker available in supplementary data (Figure 1K).

Similar to the responses observed for populations of animals in the stampede assay (Zhu et al., 2009), optomotor responses for individual wildtype CS animals display a strong optomotor response (Figure 2A). Homozygous *DopR* mutant flies for the strong hypomorph allele, *DopR^f02676^*, display poor performance in the stampede assay (Figures 2B,C). *DopR* mutant flies show a score of 29.6% in minute 1 and 29.9% in minute 2 for percent-time occupying the correct quadrant. Given that a score of 25% signifies equivalent performance to random chance for occupancy of any quadrant, *DopR* mutants display a significant deficit in their stampede optomotor response. *DopR* mutants may show less variance in their quadrant behavioral scores for the second minute optomotor performance, but the overall scores are low. The *DopR* phenotypes do suggest a slight behavioral bias for occupying both termini of the arena more than internal quadrants 2 and 3 during testing.

The DopR^f02676^ allele displays hyperactivity in response to the stampede assay conditions (Figure 2D). The mutant animals appear to travel faster than wildtype during the stampede, but this does not correlate to improved performance (Figure 2C). To investigate the parameter of animal velocity further, we measured velocity in the absence of all stimuli, and by looking at responses to the visual motion or the startle stimulus independently (Figure 2E). *DopR^f02676^* individuals did not show hyperactivity in the no stimulus or startle condition, but did travel significantly faster than wildtype when presented with visual motion or the combination of startle and visual motion that represents the standard stampede assay conditions.

Further parametric characterization of the stampede assay stimuli suggests that *DopR^f02676^* mutants are also insensitive to changes in the speed of the presented visual stimuli (Figure 3A). Whereas wildtype animals show a peak performance for visual motion at 25–40 Hz, *DopR* mutants show equivalent behavioral performance for the 0 Hz condition as compared to all other speeds. When isolating the parameter of the mechanical startle, *DopR* mutants also fail to show better performance in the presence of modulated startle (Figure 3B). Wildtype animals however display a performance peak when presented with an intermediate startle stimulus (two independent startle events: 1 s duration each with an interval of 1 s between) vs. both conditions of no startle or strong startle (three startle events, 2 s duration with 1 s intervals).

## Discussion

The stampede assay represents a rapid behavioral assay that can isolate and study sensory integration in *Drosophila melanogaster*. Given the proliferation of genetic tools and circuit manipulations in *Drosophila*, many researchers are currently focused on discovering the neural circuits and molecules used in sensory integration to create discrete behavioral decisions expressed as locomotor choices or trajectories.

Our stampede design allows for expansion of the assay for simultaneous tracking of multiple arenas. Although our design started with two chambers, it can be easily modified to allow for 6–8 independent chambers on the same footprint with the addition of two cameras in parallel. Additionally, although our population experiments utilize a long square tube (1 cm × 1 cm × 15 cm) for an arena, experimenters can easily modify arena shape to allow for better discrete tracking of populations. One disadvantage of the square tube is the higher degree of freedom for population movement through the arena, allowing for higher numbers of potential tracking conflicts for flies superimposed in space from acquiring top-down video images. Preliminary experiments with “flat” tubes (3 mm × 10 mm × 152 mm) suggest that the optomotor behavior can be observed with fewer tracking conflicts and lower fly numbers than the original population experiments (data not shown). We encourage users to try different arena designs and shapes to meet their individual needs for manipulating startle events or visual motion presentation.

Initial characterization of the stampede assay utilized populations of flies rather than individual animals (Zhu et al., 2009). Given the potential for fly-fly interactions in shaping the optomotor behavior and data suggesting a role for social contact in contributing to stable changes in locomotor pattern (Suh et al., 2004; Ramdya et al., 2015), we sought to initially investigate single-fly experiments to remove any population-based confounds in assessing optomotor responses for wildtype flies. Individual animals do display a robust optomotor response (Figure 2A) and optimal behavioral performance is positively regulated by an increased arousal state (Figure 3B). Previous experiments that placed individual animals within the large population arenas (1 cm vertical axis) did not show reliable optomotor performance (data not shown), whereas the spatial restrictions of individuals within the round 5 mm diameter tubes appear to strengthen the behavioral response. It is likely that the stronger response for individuals in the smaller 5 mm arenas is due to limiting the degrees of freedom for locomotion along the vertical axis. This may either make presentation of the visual motion stimulus more fixed for the animals’ perception based on the position of the individual, or the spatial restrictions of the tube simply bias locomotor responses more strongly along the horizontal axis of the tube than the vertical axis. The trajectory of optomotor response to visual motion is clear and the behavior is consistent, allowing for robust comparisons between genotypes or potential circuit manipulations.

While the purpose of the initial characterization of the fly stampede assay was to understand the neural circuitries that regulate optomotor and phototactic behavior (Zhu et al., 2009), we modified this assay to allow for mutant and circuit screens to understand the relevance of the change in arousal state and its positive effect on optomotor performance (Figure 3B). An individual assay is fast to perform (four independent, individual flies per trial, *n* = 4) vs. the original assay that measures the performance of an entire population of 100 animals in a single trial (*n* = 1). Preparation and execution of a single trial takes approximately 10 min. Therefore, the throughput of one afternoon of work during a set circadian period (5 h) is *n* = 120 for single fly assays, using the dual arena/single camera setup, and *n* = 30 for population assays using the original single arena setup, or *n* = 60 for our dual arena/single camera set up. This allows greater statistical power for comparison of genetic manipulations. Additionally, since our data suggests a robust and clear phenotype for individual flies (Figure 3), this also allows a higher screening efficiency since fewer genetic crosses and fewer animals are required to study the linkage to arousal and optomotor behavior. The single fly experiments also avoid confounds due to population effects due to social interactions or technical limitations caused by the inherent unreliable spatial discrimination of individual flies due to crowding or overlap in the same position that cannot be separated by the camera or tracking software.

The Yerkes-Dodson Law broadly postulates that behavioral performance on a given task is modulated by arousal state, and too little or too much arousal can prevent optimal performance. This inverted-U relation between arousal level and task performance corresponds to the “Hebbian version” of the Yerkes-Dodson law (Hebb, 1955; reviewed in Diamond et al., 2007). The stampede optomotor performance of wildtype animals in response to mechanical startle (Figure 3B) also appears to support a potential junction point in *Drosophila* sensory integration, where neural circuits that mediate startle response converge somewhere in visual circuitry to modify discrete behavioral outputs to visual motion. Previous experiments have separated neural substrates of startle-based arousal from sleep-wake arousal (Lebestky et al., 2009) and point to the Ellipsoid Body (EB) as a potential site of regulation for mechanical startle that influences expression locomotion in response to the visual motion perception and future experiments will target this region among other substrates for sensory integration.

*DopR^f02676^* mutants display poor performance in the stampede assay (Figures 2B,C) as well as hyperactivity (Figure 2D). Given a previously described role for *DopR* in hypersensitivity to mechanical startle that is expressed by long bouts of hyperactivity (Lebestky et al., 2009), it is important to consider the correlation of hyperactivity to a loss of behavioral performance in the optomotor stampede. First, this allows us to exclude the possibility that the *DopR* mutants are incapable of performing well in the optomotor response due to locomotor deficits that prevent the animal from occupying the correct quadrant before the stimulus changes or ends. Second, this raises a possibility that *DopR* animals may either perceive the visual motion differently than wildtype animals, or their hyperactivity results in an inability to maintain occupancy in the correct quadrant. This result may suggest that inhibition of excessive locomotion in response to startle may be required for appropriate visual perception. This data also suggests that arousal state, as it relates to saliency or visual-attention in the *Drosophila* brain, is modulated by dopamine. This is supported by previous studies implicating a role for dopamine in salience and visual atttention (Ye et al., 2004; Zhang et al., 2007; van Swinderen and Brembs, 2010; Koenig et al., 2016).

Simple vision assays for the avoidance of looming shadow threat suggest that *DopR* mutants are not blind to visual stimuli (data not shown), but more refined tests and measures of physiological activity in actively behaving animals will be necessary to further investigate a role for *DopR* in visual processing. Future experiments will therefore expand our initial analysis by mapping the DopR requirement in the brain using the *DopR^f02676^* allele, which contains a UAS element for restoration of DopR function when crossed to Gal4 lines (Lebestky et al., 2009; Kong et al., 2010; Pitmon et al., 2016), as well as parallel loss of function experiments utilizing the UAS-DopR-RNAi reagent to isolate the *DopR* function in discrete circuits of the adult *Drosophila* brain (Keleman et al., 2012).

Significant strides have been made in establishing *Drosophila* as a model for attentional processing (reviewed in de Bivort and van Swinderen, 2016). Behavioral studies, calcium-imaging studies, and electrophysiological correlates observed in behaving animals suggest multiple neuroanatomical loci that may contribute to selective attention, including circuits of the optic lobes, the central complex, and the mushroom body. DopR protein expression is present in many of these structures (Kim et al., 2007; Lebestky et al., 2009; Kong et al., 2010), and understanding parallel or separable roles for this molecule in the stampede behavior may facilitate deeper investigations into attentional processing.

## Author Contributions

SK, KT and GB contributed all experimental data for Figures 2, 3, and photographs for Figure 1. SK, KT and GB all contributed to elements of the article and aided TL in writing and editing the manuscript. TL wrote and edited the manuscript.

## Funding

This work was supported by the Groff Foundation and the Hellman Fellows Program.

## Conflict of Interest Statement

The authors declare that the research was conducted in the absence of any commercial or financial relationships that could be construed as a potential conflict of interest.

## Footnotes

^1^Trikinetics.com
