# Supplementary material for: Fly Stampede 2.0: A Next Generation Optomotor Assay for Walking Behavior in Drosophila Melanogaster
Source: Front Mol Neurosci. 2016 Dec 27;9:148. doi: 10.3389/fnmol.2016.00148 (PMC5214522; doi:10.3389/fnmol.2016.00148)
Supplement: Supplementary file 1 [file Software.zip › Master Folder for Fly Stampede Software_43MB/BIAS Stampede GUI Controller Software/Instructions for BIAS Stampede GUI.docx]

Instructions for BIAS Stampede GUI

The BIAS Stampede Graphical User Interface (GUI) allows the experimenter to coordinate the timing and execution of the vibration stimulus, the LED motion stimulus, and the recording of video. This integration is controlled in part by configuration files that define the ordering of events and the pattern and speed (Example included for 25 Hz LED motion stimulus with both the buzz and no buzz configurations used as the standard assay within the paper). Timing of the triggering and duration of filming is controlled within the GUI (Settings/Timer).

We have included a basic user protocol as well as a generic configuration file that can be easily modified to suit alternate experimental needs.

The zipped application and related files for the BIAS GUI are found within this folder.
