# Supplementary material for: Fly Stampede 2.0: A Next Generation Optomotor Assay for Walking Behavior in Drosophila Melanogaster
Source: Front Mol Neurosci. 2016 Dec 27;9:148. doi: 10.3389/fnmol.2016.00148 (PMC5214522; doi:10.3389/fnmol.2016.00148)
Supplement: Supplementary file 1 [file Software.zip › Master Folder for Fly Stampede Software_43MB/BIAS Stampede GUI Controller Software/User GUI Protocol/BIAS Protocol.docx]

Basic Protocol for BIAS GUI to run standard stampede assay

1) Run “bias_gui_vOp53” program

2) Select “Plugins” tab

3) Select “Settings…”

4) Go To “Load”

5) Load “Stampede Configs” from Desktop

6) Choose a configuration needed (25v is the standard assay for our experiments)

7) Select and Turn on Startle and Light Panels controller machines

8) Click “Connect All” (connects startle and light controllers)

9) Exit “Settings…”

10) Click “Timer” tab and set your desired time windows (3 mins 20 secs is the length of the entire assay)

11) Exit timer

12) Enable logging under camera.

13) Click “connect” at the bottom left (connects the camera)

14) Click start to execute the vibration/LED/acquisition directions set within the configuration file.

15) The .avi file is created to document video from the camera and the user may select a post- processing video tracking program (Ethovision or our Centroid tracker-included).
