# Supplementary material for: Fly Stampede 2.0: A Next Generation Optomotor Assay for Walking Behavior in Drosophila Melanogaster
Source: Front Mol Neurosci. 2016 Dec 27;9:148. doi: 10.3389/fnmol.2016.00148 (PMC5214522; doi:10.3389/fnmol.2016.00148)
Supplement: Supplementary file 1 [file Software.zip › Master Folder for Fly Stampede Software_43MB/Fly Population Tracker (Centroids)/Flytracking_Python_Setup/flytracking_python_setup_notes.pdf]

Python setup notes for flytracking

- Install miniconda - python package manager
  Download page http://conda.pydata.org/miniconda.html
  Select Python 2.7 version, almost certainly 64 bit version (but is based on system type)

- Create “flytracking” environment. Open command window and run the following command

  conda create -n flytracking numpy scipy matplotlib ipython

  Notes: conda is a package manager which lets you install packages into different environments.   The environments are separate and you can have different packages installed in different environments. This is really handy for development. In order to use an environment you “activate” it as follows” (type commands into command window)

  activate flytracking

  The environment - in this case flytracking - is only activated for the current command window (where you typed the commands).

  You can deactivate the environment with using the deactivate command as follows: (again run command in command window where the environment is activated). :

  deactivate

- Install opencv - it is not in the normal package location so we need a special command to install this. First open a command window and activate the flytracking environment.

  activate flytracking

  Next install opencv (in the same command window) with the following command.

  conda install -c https://conda.binstar.org/menpo opencv3

- Install a good text/code editor. Perhaps atom https://atom.io/   which is pretty nice and easy to use.

Part II

We are going to recreate the flytracking environment - just in case. I had some issues with my first version - basically some of the packages didn't seem to install correctly and I had to re-install them. So I've put together some instructions for removing the old flytracking environment and the re-installing the packages one at a time.

- Remove flytracking environment - open command window and run the following command.

  conda remove --all -n flytracking

  This should remove the flytracking environment and all installed packages.

- Create new flytracking environment with the following command.

  conda create -n flytracking numpy

  This will create the flytracking environment with the numpy package installed.

- Activate the new flytracking environment with the following command

  activate flytracking

  Note, prompt should change to be something like ((flytracking)) C:\somedirectory>

- Install the scipy (Scientific Python) package with the following command

  conda install scipy

- Install matplotlib (plotting package) with the following command

  conda install matplotlib

- Install ipython interactive command shell with the following command

  conda install ipython

- Install opencv computer vision library with the followign command

  conda install -c https://conda.binstar.org/menpo opencv3

- Install Mercurial revision control system.

  https://www.mercurial-scm.org/release/windows/Mercurial-3.8.3-x64.exe

  This will download a windows installer. Run the installer to install mercurial on your system. . Note, Mercurial (hg) is the revision control software I'm using for the stampede_tools repository - which has the background and tracking programs.

  As a test open a command window and try the command

  hg help

  This should print the basic help page for mercurial. Note, "hg" is the mercurial program.

- Create a new directory where you will keep a copy of the "stampede_tools" repository, e.g. C:\Users\Tim\stampede_repos

- Open a command window and activate the "flytracking" environment.

  activate flytracking

- In the command window change directory to the one you created above .. e.g. C:\Users\Tim\stampede_repos

  cd C:\Users\Tim\stampede_repos

- Clone "stampede_tools" repository using mercurial

  hg clone http://bitbucket.org/iorodeo/stampede_tools
  This will create a directory called "stampede_tools" in the current directory.

- Change directory to the the stampede_tools directory

  cd stampede_tools

- Install the stampede_tools package. Note, make sure the "flytracking" environment is activated - this was done in an earlier step. The prompt should be something like ((flytracking) C:\Users\ …. \stampede_tools>

  We will do a develop install - this will only put links into the site_packages directory as opposed to copying the files. This way we can make edits to the files in the repository location and this will change how the installed program functions. The installation command is

python setup.py develop

The stampede_tools software should now be installed

- Create a new directory for testing the stampede tools programs. Note, this should not be inside the stampede_tools directory. Maybe something like C:\Users\Tim\stampede_test
  This can be done with the following command

  mkdir C:\Users\Tim\stamped_test (assuming C:\Users\Tim exists … just an example)

- There is an example json parameters file in the stampede_tools\examples directory named "params.json" Copy this file to the directory made above "stampede_test" or whatever you called it.

- Also you will need a stampede video on which to run the test. I've been using

  bias_video_cam_0_date_2016_02_11_time_10_50_14_v001.avi

  This might be a good video to start with as the ROI in the params.json file will be correct.

- To run the stampe-tools command line program you will need to open a command window and activate the "flytracking" environment. Then you should cd into the testing directory created above (stampede_test). This directory should now contain the parameters file (params.json) and the video file.

  To create a background image for video - this is required for tracking - run the following command from within the stampede_test directory

  stampede-tools background params.json

  To run the tracking algorithm and output the centroid positions run the following command

  stampede-tools tracking params.json

- The basic format for calling the stampede-tools program is

stampede-tools <command> <parameters file>

Right now there are only to supported commands “background” and “tracking”.

The parameters file contains the various parameters used in finding the background model and for the tracking.

The current contents of the parameters file (params.json) is as follows

```
{
"input_video" : "bias_video_cam_0_date_2016_02_11_time_10_50_14_v001.avi",
"tracking_output_video" : "tracking_output.avi",
"background_file" : "background.png",
"median_centroid_file" : "median_centroid.txt",
"background_frame_skip" : 10,
"background_threshold"  : 90,
"background_check_roi"  : true,
"roi_cols" : [0,1280],
"roi_rows" : [350,470],
"open_kernel_size" :  [3,3],
"blob_area_min" : 10,
"blob_area_max" : 400
}
```
