# Supplementary material for: Fly Stampede 2.0: A Next Generation Optomotor Assay for Walking Behavior in Drosophila Melanogaster
Source: Front Mol Neurosci. 2016 Dec 27;9:148. doi: 10.3389/fnmol.2016.00148 (PMC5214522; doi:10.3389/fnmol.2016.00148)
Supplement: Supplementary file 1 [file Software.zip › Master Folder for Fly Stampede Software_43MB/Fly Population Tracker (Centroids)/Instructions for using centroid tracker.docx]

Instructions for using centroid tracker

This folder contains instructions for using centroid tracker for fly populations within the stampede assay. The tracker will process the .avi files to produce data that highlights the mean trajectories of all animals through the tube. Users will need to follow the download instructions within the Flytracking_python_setup_notes file. An example of the centroid data and a movie file are included. Users will also need to go to the following link to download the tracking script.

<http://bitbucket.org/iorodeo/stampede_tools>
