# Supplementary material for: Fly Stampede 2.0: A Next Generation Optomotor Assay for Walking Behavior in Drosophila Melanogaster
Source: Front Mol Neurosci. 2016 Dec 27;9:148. doi: 10.3389/fnmol.2016.00148 (PMC5214522; doi:10.3389/fnmol.2016.00148)
Supplement: Supplementary file 1 [file Software.zip › Master Folder for Fly Stampede Software_43MB/Fly Stampede Design Files /README_Stampede Design Files (Blueprints) Introduction.pdf]

Included in this folder are the design files and bill of materials files (with suppliers and 2015 prices) for purchase and production of all parts of the Fly Stampede Arena (without camera). We chose a Flea3 point grey camera but many machine vision or commercial cameras are sufficient for acquisition of video.

https://www.ptgrey.com/flea3-usb3-vision-cameras

Design Files are in multiple formats for production using pyCAD and other user formats. Interested users can use either windows or linux/MacLinux for accessing files.

If you have any additional questions about designs or modifications, feel free to contact the corresponding author (T. Lebestky at tjl3@williams.edu). Additionally, the engineers at ioRodeo are available for independent consultation for modification or production of the arena (http://www.iorodeo.com).
