# Supplementary figures and images for: Fly Stampede 2.0: A Next Generation Optomotor Assay for Walking Behavior in Drosophila Melanogaster
Source: Front Mol Neurosci. 2016 Dec 27;9:148. doi: 10.3389/fnmol.2016.00148 (PMC5214522; doi:10.3389/fnmol.2016.00148)

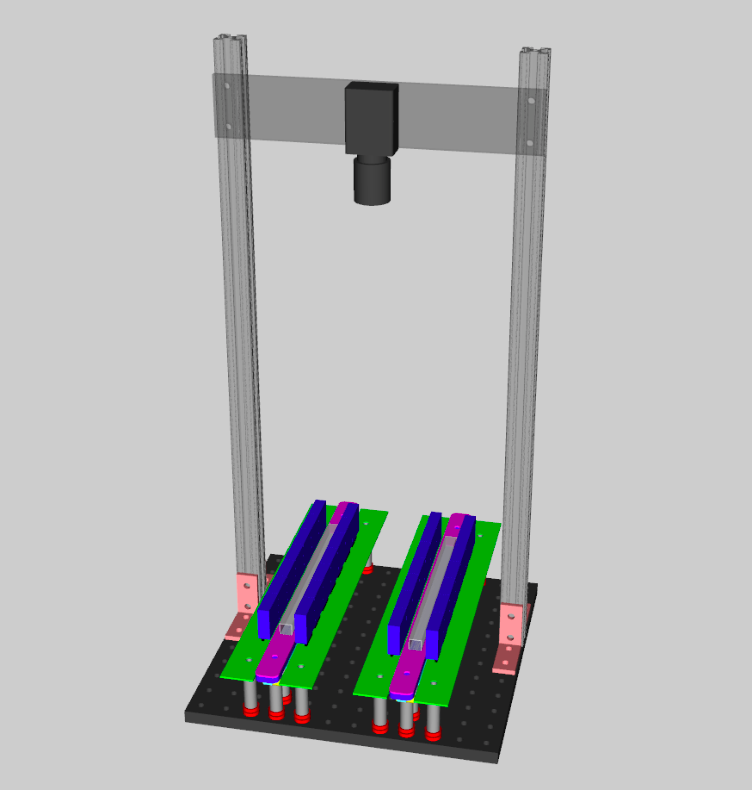

Supplement: Supplementary file 1 [file Software.zip › Master Folder for Fly Stampede Software_43MB/Fly Stampede Design Files /Cartoon_Arena.tiff]

1" reference cube

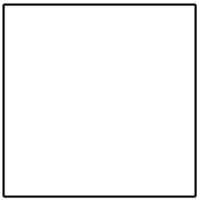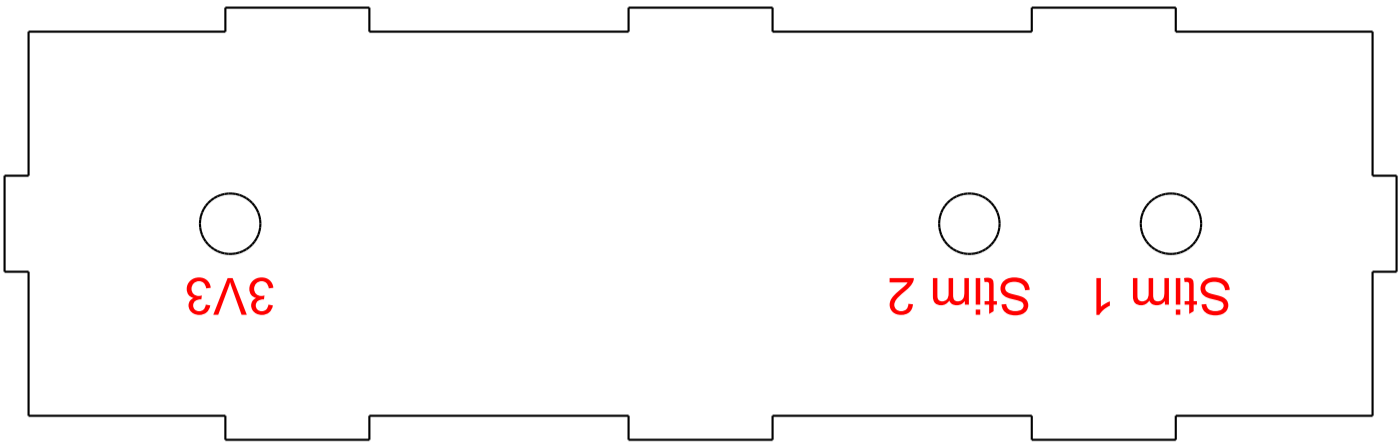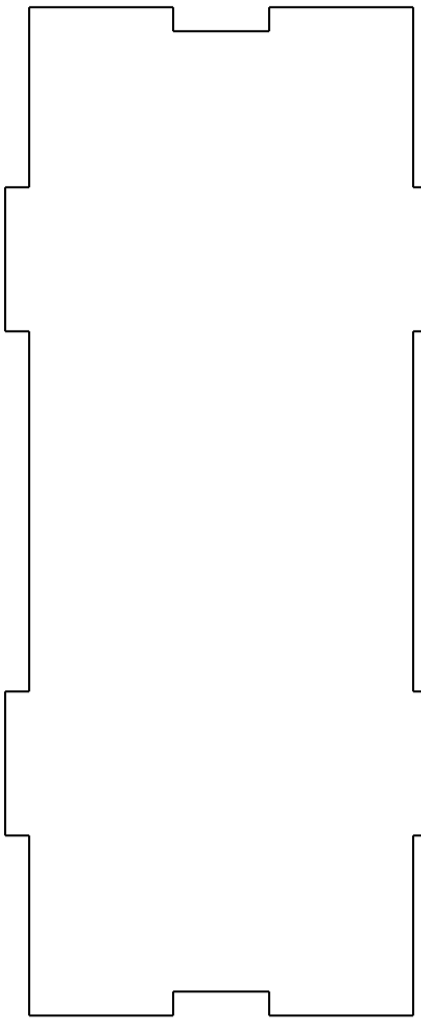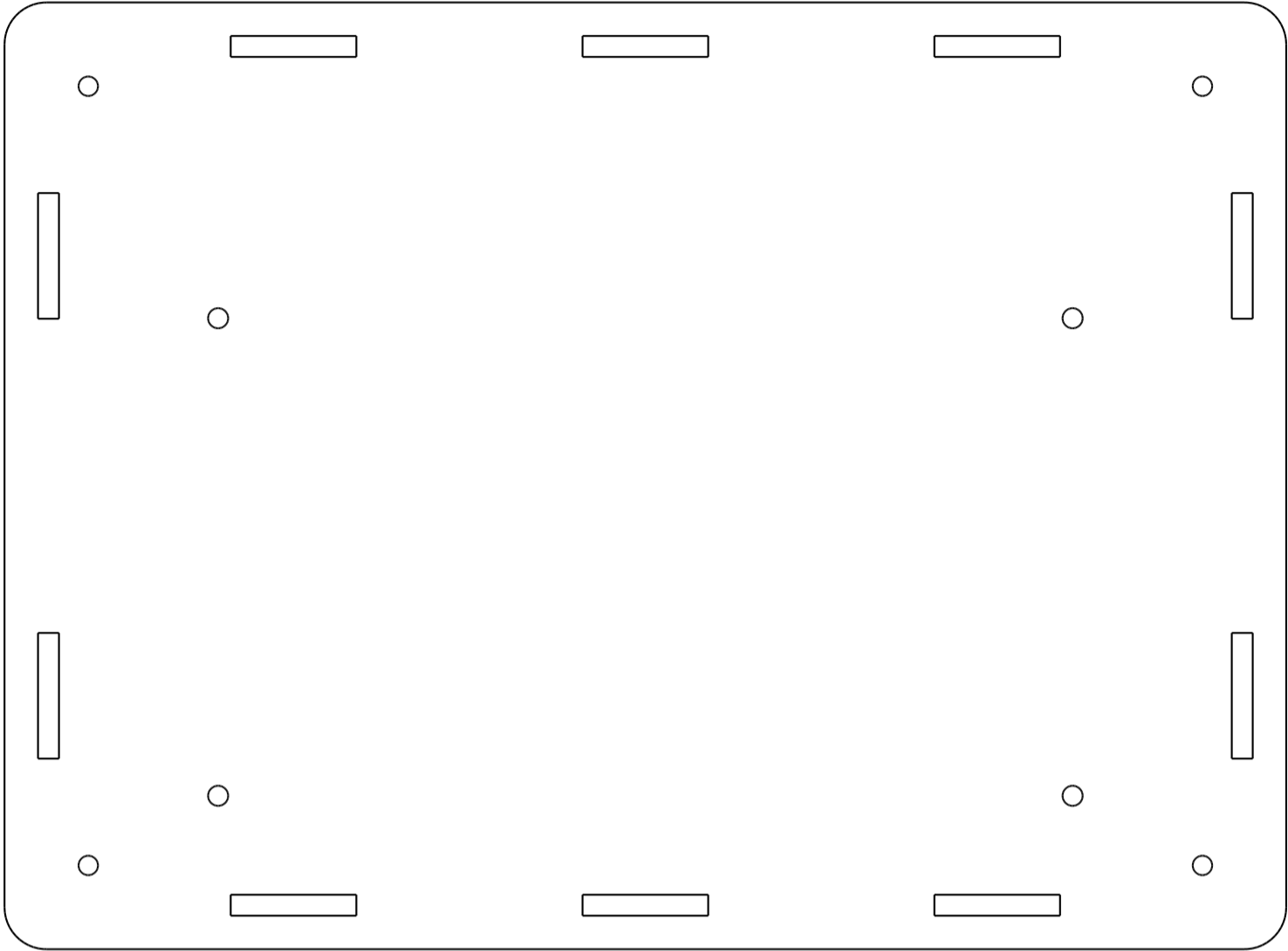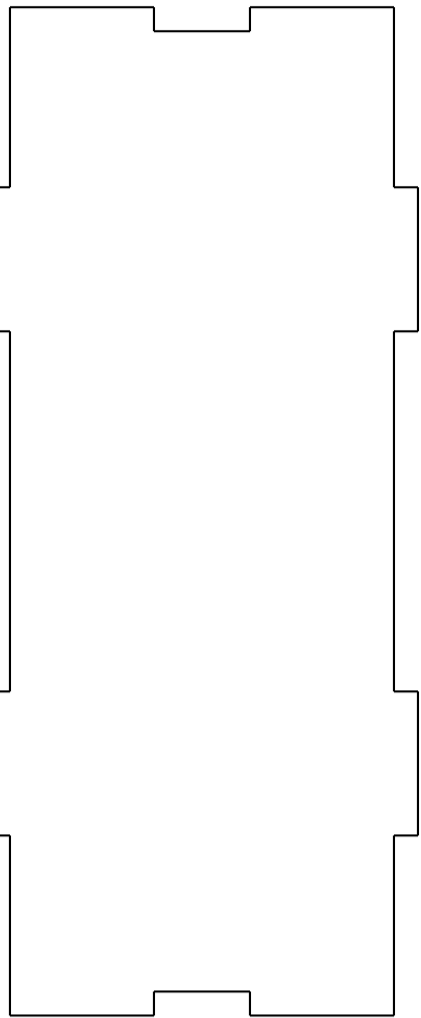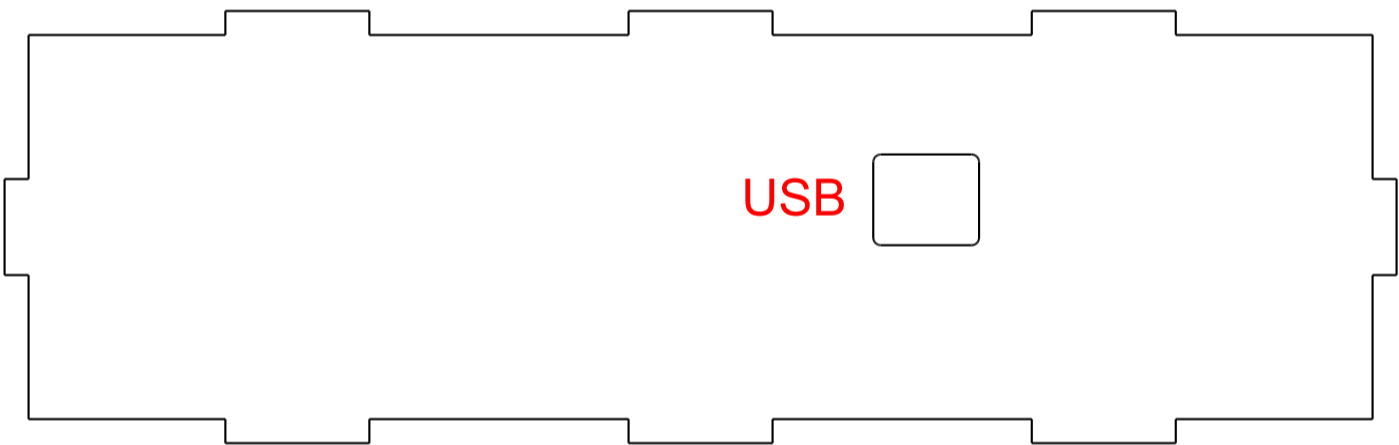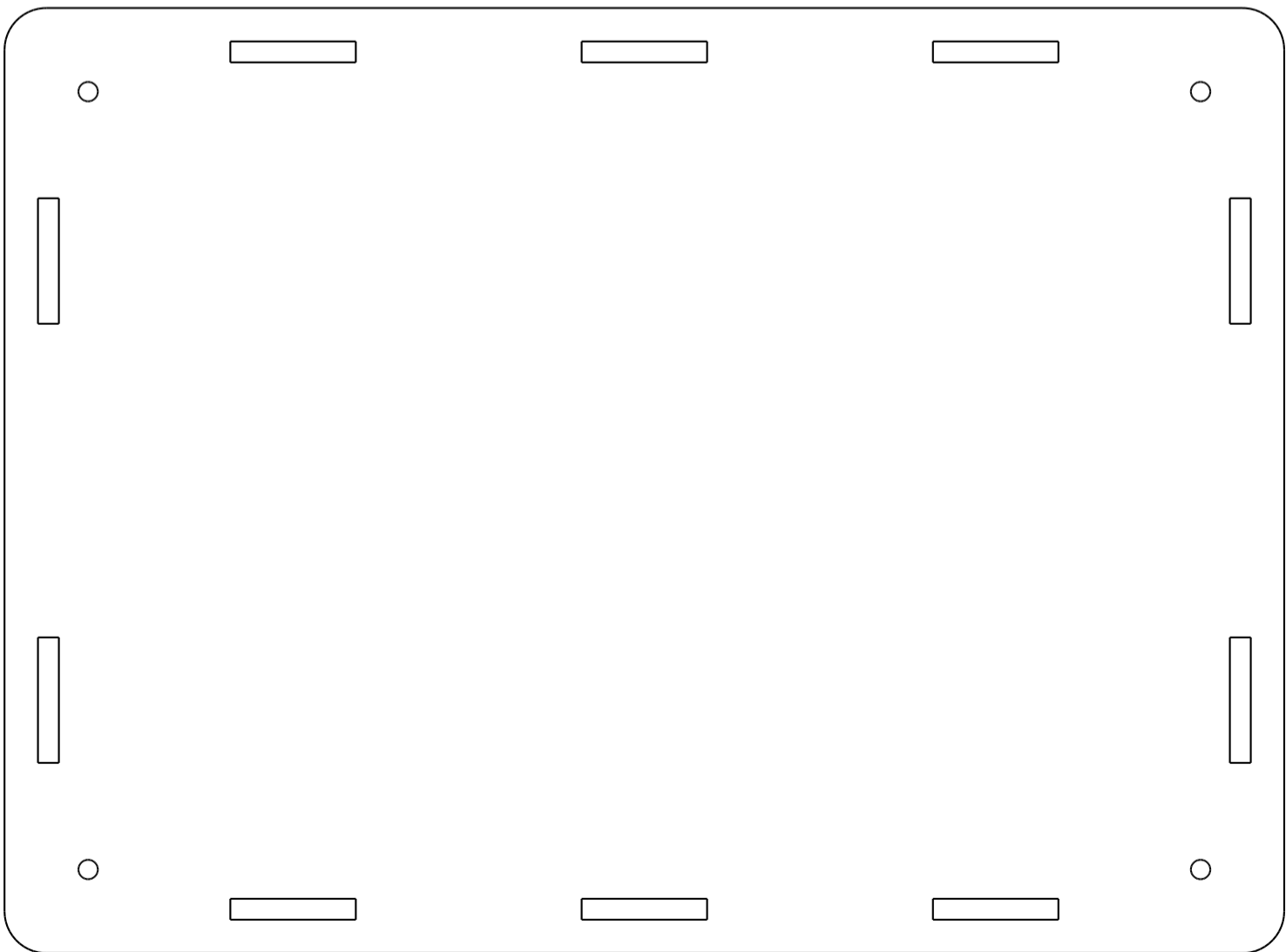

Supplement: Supplementary file 1 [file Software.zip › Master Folder for Fly Stampede Software_43MB/Fly Stampede Design Files /enclosures/nano_ssr_v1/laser_cutting_files_r1p0/enclosure_projection.pdf]

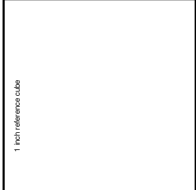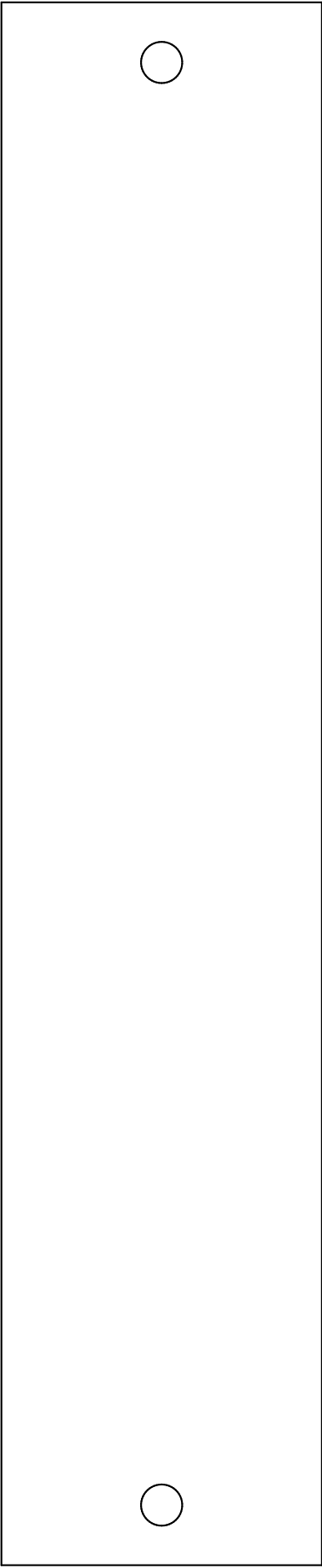

Supplement: Supplementary file 1 [file Software.zip › Master Folder for Fly Stampede Software_43MB/Fly Stampede Design Files /mechanics/dxf_version_v1p0/diffuser.pdf]
